# Arginase 1 deficiency: a treatable form of spastic paraplegia

**DOI:** 10.1007/s10072-025-08153-3

**Published:** 2025-04-16

**Authors:** Alessandro Burlina, Anna Ardissone, Roberta Battini, Alberto Burlina, Serena Gasperini, Andrea Pession, Francesco Porta, Carlo Dionisi Vici

**Affiliations:** 1 Dept. of Medicine, Neurology Unit, St. Bassiano Hospital, Via dei Lotti 40, 36061 Bassano del Grappa, Italy; 2 Child Neurology Unit, Department of Pediatric Neurosciences, Fondazione IRCCS Istituto Neurologico Carlo Besta, 20133 Milan, Italy; 3 Department of Clinical and Experimental Medicine, University of Pisa, Pisa, Italy; 4 Department of Neuroscience, IRCCS Stella Maris Foundation, Calambrone, Pisa Italy; 5 Division of Inherited Metabolic Diseases, Department of Woman’s and Child’s Health, University Hospital, 35129 Padua, Italy; 6 Department of Pediatrics, Fondazione IRCCS San Gerardo dei Tintori, Monza, Italy; 7 Pediatric Unit, IRCCS Azienda Ospedaliero-Universitaria di Bologna, 40138 Bologna, Italy; 8 Department of Pediatrics, AOU Citta’ della Salute e della Scienza di Torino, 10126 Torino, Italy; 9 Division of Metabolic Diseases and Hepatology, Bambino Gesù Childrens Hospital IRCCS, Rome, Italy

**Keywords:** Arginase 1 deficiency, Urea cycle disorder, Spastic paraplegia, Neurological impairment, Differential diagnosis

## Abstract

**Background:**

Arginase 1 deficiency (ARG1-D) is a rare hereditary urea cycle disorder characterized by elevated arginine levels, resulting in progressive neurological impairment and severe physical and cognitive disability. Due to its low prevalence, overlapping symptoms with other neurological disorders, and current limitations in newborn screening tools, ARG1-D is often misdiagnosed or diagnosed late, limiting access to early interventions.

**Aim:**

This review and expert opinion aim to provide an overview of the clinical manifestations, diagnostic challenges, and treatment options for ARG1-D, offering a practical resource for specialists to recognize this rare, progressive, yet treatable disease.

**Results:**

ARG1-D typically presents with progressive spastic paraplegia, developmental delays, cognitive impairment, and seizures, with symptom onset and severity varying by age. Differential diagnoses mainly include hereditary spastic paraplegia, cerebral palsy, and hyperornithinemia-hyperammonemia-homocitrullinuria syndrome, each with distinct clinical features and biochemical markers. Potential red flags for ARG1-D include elevated plasma arginine levels, spasticity, seizures, and cognitive impairment. These should prompt further examinations to confirm the diagnosis, which is based on biochemical assays for hyperargininemia and on genetic testing. Once confirmed, early treatment is advised, including dietary protein restriction, ammonia scavengers, antiepileptic drugs, and novel therapies, such as pegzilarginase, which targets the disease’s metabolic root.

**Conclusion:**

Experts stress the importance of increased awareness of ARG1-D characteristics, noting that early recognition and treatment are crucial to patient outcomes. Greater recognition of ARG1-D’s distinctive features, differential diagnosis, and diagnostic tools, even among non-specialist clinicians, could help prevent misdiagnoses and facilitate the identification of this rare yet treatable condition.

## Introduction

### ARG1-D etiopathology and clinical presentation

Arginase 1 deficiency (ARG1-D) is a rare hereditary metabolic disorder within the urea cycle disorder (UCD) group, caused by mutations in the *ARG1* gene [1]. Impaired arginase 1 enzyme activity leads to elevated levels of arginine and its toxic metabolites, such as guanidino compounds (GCs), in the plasma and cerebrospinal fluid, causing multiple disease manifestations that mainly affect the central nervous system [2]. Research has shown that elevated GC levels have neurotoxic effects and that injections of GC, such as guanidinoacetic acid (GAA), induce seizures and/or convulsions in rodents [3, 4]. Similar neurotoxic effects of GAA have been observed in humans, where reducing GAA levels in patients with guanidinoacetate methyltransferase deficiency was associated with fewer seizures, highlighting GAA’s role as a neurotoxin [5, 6].

**Fig. 1:**
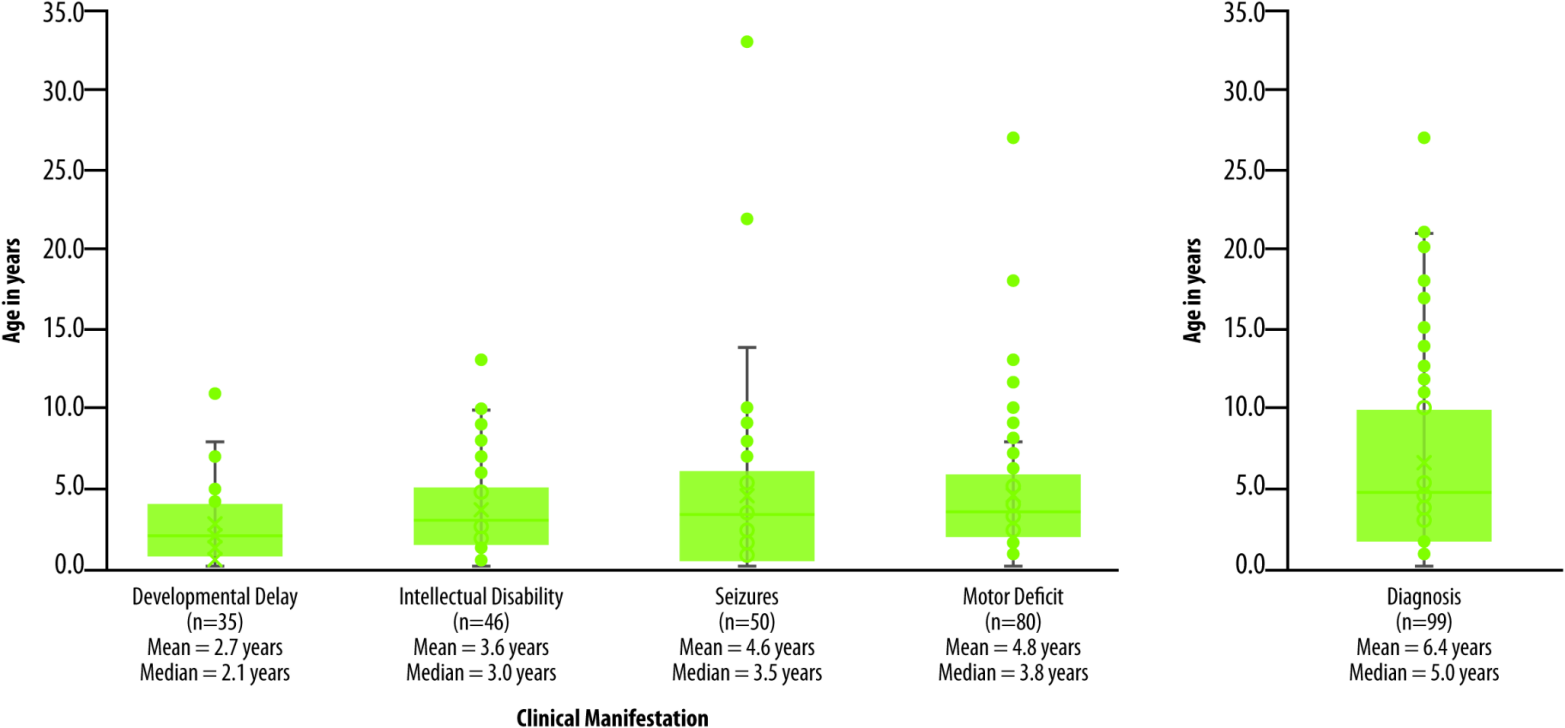
Age of onset of clinical manifestations and diagnosis. Adapted from Bin Sawad 2022 [7]; published open access under the terms of the Creative Commons Attribution License

Neurological manifestations in ARG1-D patients typically include progressive spastic paraparesis, developmental delay, cognitive impairment, and seizures, which vary in different onset’s age (Fig. 1) [7]. Clinical onset in pediatric age is generally characterized by neurological symptoms such as clumsiness, increased frequency of tripping and falling, and growth delay [8]. Without timely treatment, these neurological symptoms progress to pronounced spasticity and gradual regression of developmental milestones. ARG1-D may also impair cognitive functioning, with the Full-Scale Intelligence Quotient (FSIQ) lower than the general population average, contributing to poor school outcomes [9]. Behavioral issues represent another hallmark of the condition. Besides development regression, patients with ARG1-D often experience short-term memory loss and a progressive decline in adaptive behavior. Behavioral problems include irritability, hyperactivity, inability to follow commands, and lack of concentration [8, 10–14].

In addition to severe neuromotor symptoms and intellectual disabilities, patients may experience complications arising from inadequate nutrition, growth disorders, liver cell damage, and hyperammonemia. Although the age of onset and the rate of progression vary among individuals, no clear correlation has been established between age and clinical manifestations. However, the overall clinical burden in these patients tends to increase over time, potentially leading to a higher risk of early death [8, 15–18]. For example, in a cohort of 140 individuals, the median age at death was reported as 17 years [19].

### ARG1-D diagnosis and current limitations

Diagnosis of ARG1-D is challenging because of the extreme rarity of the disease, with an estimated prevalence of 1:726.000-950.000 worldwide [20, 21]. In Italy, its prevalence is estimated at 0.61 cases per million, with 36 cases estimated in 2021 in a population of 59,038,000 [20].

Although multiple tools are available for diagnosing ARG1-D, including clinical assessments, biochemical analyses, and genetic testing, delays in diagnosis or misdiagnosis remain common. Such delays negatively impact the access to existing treatment options for patients [1, 20].

In terms of genetic variants, the most common mutations in *ARG1* deficiency are the missense mutations p.Thr134Ile and p.Gly235Arg, and the nonsense mutation p.Arg21*, which are geographically clustered in Brazil, China, and Turkey [22]. However, novel variants have also been reported in case studies, including Pakistani [23], Sudanese [24], Chinese [25], and Japanese patients [26].

Newborn screening (NBS) for ARG1-D, which relies on quantifying arginine levels from dried blood spots (DBS), serves as a valuable diagnostic tool despite some limitations. First, ARG1-D is not universally included in all NBS panels worldwide. Furthermore, establishing an accurate diagnostic cut-off to reliably assess arginase deficiency is challenging because of the variations of arginine in neonates. As a result, NBS based solely on arginine level detection in DBS, without supplementary tests (e.g., genetic testing or arginase activity in red blood cells) may lead to false-negative results, failing to identify ARG1-D during the neonatal period [1, 27]. The potential utility of the arginine/ornithine ratio has also been evaluated for NBS [28].

In Italy, expanded metabolic NBS among 806,770 infants screened between January 2019 and December 2020 detected one newborn with ARG1-D, corresponding to an incidence of 1 in 806,770 [29].

Undiagnosed patients with ARG1-D are likely to develop progressive symptoms with increasing age and are typically referred to metabolic pediatricians, pediatric neurologists, pediatric neuropsychiatrists, or adult neurologists, depending on their age and symptomatology. Unfortunately, due to the extreme rarity of ARG1-D, many healthcare professionals often lack awareness of the condition [1, 20]. Furthermore, the diverse clinical presentation of ARG1-D overlaps with other conditions, such as cerebral palsy (CP) and hereditary spastic paraplegia (HSP), leading to a risk of misdiagnosis and potentially inappropriate treatments [30].

### ARG1-D personal and healthcare burden

Patients with ARG1-D face lifelong challenges and require early and increasingly intensive healthcare support as the disease progresses [31]. They often utilize more healthcare resources than the general population because of the severity and frequency of symptoms, including spasticity, developmental delays, intellectual disabilities, and seizures [7]. In particular, patients require dietary protein restriction and pharmacological treatments, such as ammonia scavengers, to manage metabolic imbalances [2]. Additional therapies include antiepileptic medications, spasticity management (e.g., muscle relaxer drugs, botulinum toxin injections, and physiotherapy), and frequent hospitalizations. Regular monitoring is essential to assess the metabolic profile (e.g., ammonia levels, blood amino acids), liver function, spasticity, and developmental progress [32]. With disease progression, patients may increasingly rely on assistance for daily activities, as they often lose independence in performing essential tasks such as eating, speaking, reading, and walking [12]. Finally, patients with ARG1-D are at higher risk of developing comorbid conditions, particularly liver diseases, including cirrhosis and fibrosis, which necessitate appropriate monitoring and treatment [7].

Managing the complex clinical picture in ARG1-D imposes substantial societal costs and significantly impacts health-related quality of life. Early diagnosis and improved treatment options that delay or halt disease progression may have the potential to enhance the quality of life for patients, caregivers, and impact on societal costs [18].

In this paper, we review the current knowledge and expert opinions on ARG1-D, highlighting the main red flags of the disease and focusing on neurological symptoms that overlap with other conditions in both pediatric and adult populations. The aim is to provide specialists who may encounter such patients in their clinical practice with a practical tool for identifying this rare, progressive, yet treatable disease.

## Clinical presentation and differential diagnosis

### Clinical findings

A summary of the main disease manifestations in children and adults with ARG1-D is provided in Table 1.

**Table 1:** Clinical manifestations in pediatric and adult patients with ARG1-D

Clinical Manifestation	Specialist	0–6 months	6–12 months	Childhood	Adolescent	Adult
Seizures	PED/NPI/NEU	(+)	(++)	(++) Usually generalized tonic-clonic or clonic	(++) Usually generalized tonic-clonic or clonic	(++)
Spasticity	PED/NPI/NEU	-	(+)	(++) Tip-toe walking	(+++) Progressive spasticity	(+++) Progressive spasticity
Neuromotor and cognitive skills	PED/NPI	-	(+)	(++) Global delay or regression after initial normal development	(++) Gait impairment Loss of spoken language Loss of sphincter control Polyneuropathy	(++) Gait impairment Loss of spoken language Loss of sphincter control Polyneuropathy
Impaired mobility/balance	PED/NPI		(+)	(++)		
Slowdown of the growth process	PED			(+)	(+)	
Spontaneous avoidance of high-protein foods	PED/NPI		(++)	(++)		
Episodes of mild hyperammonemia*	PED/NPI	(+)	(+)	(+)		
Hepatic pathology	PED/NPI/NEU			(+) Liver fibrosis, acute liver failure	(+) Liver fibrosis, acute liver failure	(+) Occasionally hepatocellular carcinoma

NEU: neurologist; NPI: pediatric neurologist; PED: pediatrician

*Encephalopathy, irritability, feeding difficulties with poor appetite, meat aversion, nausea/vomiting

#### Pediatric patients

Although the clinical manifestations of ARG1-D vary among individuals, most patients have normal development from birth to toddlerhood, with clinical onset between 1 and 3 years of age after linear growth [31]. Similar to other UCDs, patients may experience episodic hyperammonemia of varying degrees, accompanied by elevated glutamine levels and symptoms, such as vomiting, lethargy and altered mental status [2]. Unfortunately, hyperammonemia might also be asymptomatic and can only be detected through blood ammonia measurement during acute illness [31]. Other potential indicators of ARG1-D include growth deceleration, which usually leads to growth deficiency [31] and feeding issues due to the spontaneous avoidance of high-protein foods [8, 30, 33]. Hepatic pathologies, such as neonatal cholestasis, acute liver failure, or liver fibrosis, may also occur [17].

From a neurological point of view, the main clinical features include developmental delay or regression, cognitive decline, spastic paraparesis, and epilepsy. Symptomatic patients with neurological symptoms are commonly referred to pediatric neurologists or pediatric neuropsychiatrists for evaluation. According to clinicians, a critical early indicator of the disease is a medical history of normal psychomotor development in the first months of life, followed by cognitive and motor regression after 1 year of age [30, 31]. Another common neurologic feature is lower limb spasticity, mainly tip-toe walking, which usually develops between 2 and 4 years old and is frequently misdiagnosed as CP [7, 31]. Unlike in adulthood, spasticity in pediatric ARG1-D patients may also involve the upper limbs, leading to spastic quadriplegia. In addition, patients may present with other general motor deficits, such as reduced mobility/gait and impaired balance/ataxia [7]. Contractures or lordosis are potential complications resulting from severe spasticity [31].

Seizures are relatively common, occurring in approximately 50% of pediatric patients with ARG1-D. Studies have reported both generalized and focal seizure types, including generalized tonic-clonic, tonic, atypical absence seizures, and focal motor seizures with or without impaired awareness, some of which are fever-induced. Furthermore, there are cases of non-convulsive status and epilepsia partialis continua. Among these, generalized seizures appear to be the most frequent [34]. Finally, microcephaly has also been reported in a growing number of patients [7, 35].

Coagulation disturbances have been also reported in younger patients, primarily manifested as prolonged prothrombin time (PT) and increased international normalized ratio (INR) and low levels of factor VII and IX. These disturbances may also manifest with minor bleeding, such aspetechia and ecchymosis [36].

#### Adult patients

Adult patients with ARG1-D are usually referred to a neurologist because of symptoms, such as spasticity, impaired mobility, gait impairment, seizures, or declines in cognitive functions. In particular, spastic paraplegia is a primary neurologic feature of ARG1-D in adulthood and should be carefully evaluated by clinicians. In contrast, spastic quadriplegia and ataxia are less common. Additionally, recent case reports have described occurrences of polyneuropathy [33, 37].

Adult patients with ARG1-D frequently experience varying degrees of decline in neuromotor and cognitive abilities, including loss of gait, decreased vocabulary or speech impairments, and loss of sphincter control [30, 31, 33]. Cognitive assessments often reveal IQs in the 70s, with approximately half of the patients maintaining independent living despite significant memory and fine motor deficits [38]. Some mildly affected individuals may even retain employment [31].

Seizures are also prevalent in adult patients with ARG1-D, with widely variable age of onset, ranging from as early as 4 months to as late as 30 years of age. Seizure activity often correlates with elevated levels of arginine and its metabolites [22, 39].

Finally, as in pediatric cases, adults with ARG1-D may also exhibit hyperammonemia and hepatic dysfunction, with occasional reports of hepatocellular carcinoma [31].

### Differential diagnosis

Due to the characteristic presentation of lower-limb spasticity, the gradual onset, non-specific symptoms, and the slow progression of additional signs, ARG1-D may initially mimic other neurological conditions, such as CP or HSP [40]. Furthermore, ARG1-D must be differentiated from other inherited metabolic diseases, particularly hyperornithinemia-hyperammonemia-homocitrullinuria (HHH) syndrome, which is another UCD [41, 42].

A summary of the main differential diagnoses in children and adults with ARG1-D is provided in Table 2. The main signs and symptoms distinguishing ARG1-D from CP and HSP, as well as ARG1-D from HHH syndrome, are detailed in Tables 3 and 4, respectively.

**Table 2:** Main differential diagnosis in patients with ARG1-D

Hereditary spastic paraplegia
Cerebral palsy
Hyperornithinemia-hyperammonemia-homocitrullinuria syndrome
Adreno myeloneuropathy
Mitochondrial encephalopathies
Pyrroline-5-carboxylate synthase deficiency
Cerebrotendinous xanthomatosis
Lysosomal storage disorders, especially gangliosidosis and Niemann-Pick disease type C
Genetic epileptic encephalopathy (Rett-like syndromes)

**Table 3:** Main signs and symptoms of ARG1-D vs. CP and HSP

	ARG1-D	CP	HSP
Lower-limb spasticity	√	√	√
Progression of cognitive and motor symptoms	√	No	√ (mainly in the adult forms)
Seizures	√	√	No
Alterations in arginine levels	√	No	No
Hyperammonemia episodes	√	No	No
Avoidance of protein-rich food	√	No	No
Risk factors for hypoxia during birth or the neonatal period	No	√	No
Urinary urgency	No	No	√
Specific imaging patterns	No	√	√

**Table 4:** Differential diagnosis of ARG1-D and HHH syndrome

	ARG1-D	HHH syndrome
Biochemical profile	Elevated arginine levels in plasma and cerebrospinal fluid + accumulation of guanidino compounds	Elevated plasma ornithine levels + urinary excretion of homocitrulline and often orotate as well
Hyperammonemia	Episodic hyperammonemia, with less elevated peak of ammonia levels	Episodic hyperammonemia
Spasticity	Progressive worsening of spasticity	Spasticity more gradually progressing and preceded by hyperreflexia and other pyramidal signs
Cognitive impairment	Progressive symptoms up to potentially early death	Progressive (especially in adult forms)
Seizures	Present	Present
Avoidance of high-protein food	Present	Present
Urinary urgency	Absent	Present

#### Cerebral palsy

CP is a group of disorders that typically manifest in early childhood as a result of non-progressive brain disturbances during fetal or infant development [43]. Patients with CP experience motor symptoms, such as spasticity, weakness, hypotonia, and involuntary movements, frequently accompanied by various non-motor neurological features, including intellectual disability, behavioral symptoms and seizures [43].

Several distinguishing features may help differentiate ARG1-D from CP. These include a detailed medical history that reveals progressive symptoms, such as worsening spasticity, cognitive and language decline, and spontaneous avoidance of high-protein foods, as well as the absence of risk factors for hypoxia during birth or the neonatal period [12]. Conversely, patients with CP do not exhibit abnormal arginine and ammonia levels observed in ARG1-D nor show signs of disease progression. Additionally, CP is associated with a medical history of prenatal/neonatal complications [43].

#### Hereditary spastic paraplegia

HSP comprises a group of genetic disorders characterized by predominantly lower-extremity weakness and spasticity. Additional neurological features include hyperreflexia and extensor plantar responses, with some patients reporting mildly impaired vibration sensation in the distal lower extremities [44]. The onset of HSP in early childhood may result in non-progressive symptoms resembling spastic diplegic CP. In contrast, onset in later childhood or beyond usually leads to slow, steady progression until a functional plateau is reached [44]. Interestingly, more than 80 genetic types of HSP have been identified, which can be distinguished through multigene panels [44, 45].

Key distinctions between ARG1-D and HSP include the prevalence of seizures, which are common in ARG1-D but less frequent in HSP; pronounced worsening of spasticity in ARG1-D, which is absent in HSP; possible dietary avoidance of high-protein foods, observed in ARG1-D but not in HSP; and the occurrence of urinary urgency associated with lower limb spasticity in HSP, contrasting with rare hypertonic bladder issues in ARG1-D [12, 44]. Moreover, specific MRI patterns can offer valuable clues in the diagnosis of certain HSP [40].

Finally, while patients with ARG1-D exhibit hyperargininemia and may present with hyperammonemia, these biochemical hallmarks are absent in HSP. For this reason, a definitive diagnosis typically requires biochemical or genetic testing. Experts recommend routine assessment and the inclusion of the *ARG1* gene in molecular panels for the differential diagnosis of HSP [40].

#### Hyperornithinemia-hyperammonemia-homocitrullinuria syndrome

HHH syndrome is a rare disorder of the urea cycle and ornithine degradation pathway that often leads to severe neurological symptoms, including pyramidal and cerebellar signs, movement disorders, dystonia, and epilepsy. Although HHH syndrome shares many clinical similarities with ARG1-D, key distinctions exist. Patients with HHH syndrome usually exhibit an aversion to protein-rich foods and respond well to a low-protein diet. However, unlike ARG1-D, HHH syndrome is frequently characterized by acute intermittent episodes of hyperammonemia accompanied by ataxia, vomiting, lethargy, and confusion. Such episodes are quite rare in ARG1-D patients. HHH syndrome is commonly diagnosed in childhood, whereas ARG1-D diagnosis is often delayed until adulthood [41, 42, 46, 47].

#### Other inherited metabolic diseases

Other inherited metabolic diseases that should be carefully considered in the differential diagnosis of ARG1-D include:

- Adrenomyeloneuropathy.
- Mitochondrial encephalopathies.
- Pyrroline-5-carboxylate synthetase deficiency [48].
- Cerebrotendinous xanthomatosis.
- Lysosomal storage disorders, especially gangliosidosis, Niemann-Pick disease type C.
- Other genetic (non-metabolic) diseaes: genetic epileptic encephalopathy (Rett-like syndromes).

## From red flags to diagnosis of ARG1-D

Once a physician detects potential red flags of ARG1-D, such as spasticity, seizures, and cognitive impairment, various assays can be performed to establish a diagnosis. Elevated plasma arginine levels, a hallmark of ARG1-D, serve as a primary diagnostic indicator, with concentrations approximately three- to fourfold higher than the upper limit in affected patients [31]. However, in some cases, patients may exhibit only a slight increase in plasma arginine levels. Besides arginine levels, the diagnosis can be confirmed by detecting deficient arginase activity in red blood cells and identifying biallelic pathogenic variants in the *ARG1* gene [7, 31].

Given its relatively high sensitivity, *ARG1* molecular genetic testing is the preferred confirmatory test for ARG1-D [31]. This process includes single-gene testing to identify various mutation types, such as deletions/insertions, missense, nonsense, and splice site variants, in the sequence of *ARG1*, followed by gene-targeted deletion/duplication analysis to detect intragenic deletions or duplications. Alternatively, ARG1-D can be assessed using multi-gene panels, which offer broader coverage and minimize the likelihood of identifying variants of uncertain significance in unrelated genes [31].

Additional biochemical assays supporting the diagnosis of ARG1-D include measuring arginine/glutamine, arginine/citrulline, or arginine/ornithine ratios through plasma quantitative amino acid assays. These analyses are particularly useful for distinguishing ARG1-D from other conditions, such as HHH syndrome [41, 43, 47]. Blood ammonia levels should also be determined as part of the diagnostic work-up. However, elevated ammonia levels alone are not sufficient for diagnosis. Patients with ARG1-D usually exhibit normal or mildly increased ammonia levels, although relevant elevations (e.g., concentration > 150 µmol/L) may occur during a hyperammonemia episode. Such episodes are rare in ARG1-D patients, and peak ammonia levels are generally lower than those observed in other UCDs [17]. Lastly, urinary orotic acid excretion can provide additional diagnostic support [31, 49].

Neuroimaging findings in patients with ARG1-D are limited and not specific, rendering them unreliable as diagnostic tools. Reported brain MRI findings in ARG1-D include variable white matter disorders, such as microstructural alterations in corticospinal tracts studied via diffusion tensor imaging [50], variable degrees of brain atrophy, mild cerebellar atrophy, thinning of the corpus callosum, and basal ganglia infarcts [51, 52].

## ARG1-D treatment options

Current guidelines for the treatment of ARG1-D emphasize early diagnosis and intervention to manage the condition, which, unlike other forms of spastic paraplegia, is a treatable disease. While other forms of spastic paraplegia lack specific therapies and rely solely on symptomatic treatment to alleviate muscle spasticity and enhance strength and gait [53], patients with ARG1-D have access to several treatment options aimed not only at mitigating symptoms but also at reducing plasma arginine levels, addressing the etiologic cause of the disease [49].

The standard of care for ARG1-D consists of a combination of dietary and pharmacological strategies.

According to guidelines for the management of UCDs, the main objective in ARG1-D is to lower plasma arginine levels below 200 µmol/L [2]. However, even with strict dietary restrictions, achieving and maintaining plasma arginine levels below treatment recommendations is rarely successful and can only be achieved in less severe cases [16, 17, 22, 54–56]. Challenges in meeting treatment goals stem from poor adherence to restrictive diets, which greatly impact patients’ quality of life, and the inability to fully counteract endogenous arginine production.

Dietary restriction may be combined with dietary supplementation to provide essential amino acids without increasing arginine levels. Ammonia scavengers, such as sodium or glycerol phenylbutyrate and sodium benzoate, are also used to remove excess nitrogen from the bloodstream, thereby aiding in controlling plasma arginine levels [2]. Notably, while adhering to established guidelines for treating ARG1-D, physicians may also implement additional strategies based on their clinical expertise and individual patient characteristics. For instance, some clinicians recommend using glycerol phenylbutyrate as a scavenger agent to aid in disease management. However although this agent helps reduce nitrogen levels, it does not directly lower plasma arginine and is therefore not effective in addressing the underlying pathophysiology of ARG1-D.

Liver transplantation has also been explored as a potential approach to improve the quality of life of patients with ARG1-D prior to development of neurological symptoms. A case study reported that a Japanese pediatric patient who underwent liver transplantation at the age of 1 year and 5 months exhibited normal neurodevelopment with no major medical problems up to the age of 14 years old [57].

Finally, pharmacological treatments targeting disease manifestations, such as anti-epileptic and anti-spasmodic drugs, are employed to manage seizures [2, 58].

Most recently, a novel human arginase 1 enzyme called pegzilarginase has been approved in Europe. This modified enzyme has enhanced catalytic activity and an extended half-life, contributing to reduced plasma arginine levels in patients with ARG1-D. A phase III, randomized, placebo-controlled trial showed that pegzilarginase is well-tolerated in patients with ARG1-D and effectively normalizes plasma arginine levels while achieving clinically meaningful improvements in functional mobility [59]. This innovative option may help patients achieve the current treatment goal of arginine levels below 200 µmol/L, which is rarely attainable through rigorous dietary restriction alone [31].

### Long-term prognosis

The prognosis and long-term outcomes of patients with ARG1-D are not well established. The disease is characterized by progressive spastic paraplegia and seizure control remains challenging to achieve. While data from the literature report that some patients survive into adulthood, the natural history of the disease comprises a multitude of severe clinical manifestations, such as developmental delay, intellectual disability, lower limbs spasticity, impaired mobility and seizures [1, 7].

Although ARG-1 deficiency may have a comparatively less severe prognosis compared to other urea cycle disorders, due to the relatively low levels of hyperammonemia with less frequent episodes of metabolic decompensation and subsequent brain damage [60], the accumulation of neurotoxic guanidino compounds in the plasma and CSF, caused by elevated plasma arginine, exerts neurotoxic effects, contributing to seizure susceptibility, and putting ARG1-D patients at risk for early mortality [61].

Lastly, no long-term data are currently available on patients with ARG1-D treated with pegzilarginase; and further research is needed to assess its impact on long-term prognosis and outcomes.

## Expert opinion

This expert opinion reflects the consensus of a multidisciplinary panel comprising neurologists, pediatricians, pediatric neurologists, and specialists in metabolic disorders. The panel members have substantial clinical experience in the diagnosis and treatment of patients with rare metabolic diseases, including patients diagnosed and treated for ARG1-D, which is notably the rarest disease among urea cycle disorders. Their expertise is further evidenced by their contributions in different publications in metabolic and neurological journals, including international guidelines for the diagnosis and management of urea cycle disorders.

The experts, all of whom are co-authors of this paper, conducted a comprehensive review of the literature, including research articles, clinical guidelines, and expert consensus documents, focusing on the clinical presentation, differential diagnosis, and treatment options for arginase 1 deficiency. Evidence from the literature was critically evaluated and discussed by the experts within an expert panel meeting, gathered in Bologna (Italy) in May 2024. During this meeting, the panel synthesized their opinions through structured discussions. These discussions were followed by in-depth deliberations to align and finalize the expert consensus.

## Conclusion

As ARG1-D is a treatable disease, early identification of affected patients is essential. Spastic paraplegia, either isolated or associated with other neurological signs, is a clinical hallmark of the disease, representing the target for selective screening programs. Misdiagnosis can result in ineffective or potentially harmful treatments that may negatively impact long-term patient outcomes. For instance, valproic acid, an antiepileptic medication commonly used to treat seizures, can be detrimental in patients with ARG1-D, increasing the risk of hyperammonemia [34].

Experts acknowledge several limitations in diagnosing ARG1-D. The rarity of the disease and lack of awareness among non-specialists often hinder its consideration as a potential diagnosis in patients with neurological symptoms. Additionally, overlapping symptoms with more common neurological diseases further complicate the recognition of ARG1-D patients.

Experts also acknowledge that NBS for ARG1-D has some limitations, in particular the lack of universal availability of arginine in the NBS panel and the lack of consistency in arginine cut-off values in the first days of life. The use of appropriate arginine and arginine/ornithine ratio cut-offs set on the basis of normal newborns population (and/or newborns with ARG1-D) may improve newborn screening effectiveness, with anticipation of treatment to lower plasma arginine levels [27].

Moreover, certain diagnostic tests for ARG1-D can be challenging for non-specialists to interpret. Genetic analysis, in particular, requires the expertise of geneticists to assist specialists in understanding the findings. Geneticists must be informed about the importance of incorporating the *ARG1* gene into their analyses for suspected patients and ensuring that ARG1-D is consistently included in multigene panels designed for conditions such as HSP. A multidisciplinary approach involving neurology and genetics is crucial to promptly identify this rare disorder.

In conclusion, ARG1-D is a highly impactful metabolic disorder, where diagnosis remains challenging because of its rarity and overlapping symptoms with other neurological conditions. Raising awareness of the distinctive features of the disease, main differential diagnoses, and available diagnostic tools, particularly among specialists not experts of ARG1-D, is essential to facilitate early identification of this rare and treatable condition. Multiple treatment options are available to manage ARG1-D, including dietary restrictions, ammonia scavengers, anti-seizure medications, and, most recently, innovative therapies, such as pegzilarginase, the first pharmacological treatment which is able to reduce in an efficacious manner the circulating levels of arginine. Early recognition and timely treatment are crucial for addressing the disease and improving patient outcomes.
